# Metabolomics Exploration of Pseudorabies Virus Reprogramming Metabolic Profiles of PK-15 Cells to Enhance Viral Replication

**DOI:** 10.3389/fcimb.2020.599087

**Published:** 2021-01-29

**Authors:** Hongchao Gou, Zhibiao Bian, Yan Li, Rujian Cai, Zhiyong Jiang, Shuai Song, Kunli Zhang, Pinpin Chu, Dongxia Yang, Chunling Li

**Affiliations:** ^1^ Institute of Animal Health, Guangdong Academy of Agricultural Sciences, Guangzhou, China; ^2^ Guangdong Provincial Key Laboratory of Livestock Disease Prevention, Guangzhou, China; ^3^ Guangdong Open Laboratory of Veterinary Public Health, Guangzhou, China; ^4^ Scientific Observation and Experiment Station of Veterinary Drugs and Diagnostic Techniques of Guangdong Province, Guangzhou, China

**Keywords:** metabolomics, metabolic activity, pseudorabies virus (PRV), variant virulent strain, classical attenuated strain, PK-15 cells

## Abstract

For viral replication to occur in host cells, low-molecular-weight metabolites are necessary for virion assembly. Recently, metabolomics has shown great promise in uncovering the highly complex mechanisms associated with virus-host interactions. In this study, the metabolic networks in PK-15 cells infected with a variant virulent or classical attenuated pseudorabies virus (PRV) strains were explored using gas chromatography-mass spectrometry (GC-MS) analysis. Although total numbers of metabolites whose levels were altered by infection with the variant virulent strain or the classical attenuated strain were different at 8 and 16 h post infection (hpi), the predicted levels of differential metabolic components were shown to be associated with specific pathways, including glycolysis as well as amino acid and nucleotide metabolism. The glucose depletion and glycolysis inhibitors 2DG and oxamate could reduce the level of PRV replication in PK-15 cells. In addition, the inhibition of the pentose phosphate pathway (PPP) resulted in an obvious decline of viral titers, but the prevention of oxidative phosphorylation in the tricarboxylic acid (TCA) cycle had a minimal effect on viral replication. Glutamine starvation resulted in the decline of viral titers, which could be restored by supplemental addition in the culture media. However, inhibition of glutaminase (GLS) activity or the supplement of 2-ketoglutarate into glutamine-deleted DMEM did not alter PRV replication in PK-15 cells. The results of the current study indicate that PRV reprograms the metabolic activities of PK-15 cells. The metabolic flux from glycolysis, PPP and glutamine metabolism to nucleotide biosynthesis was essential for PRV to enhance its replication. This study will help to identify the biochemical materials utilized by PRV replication in host cells, and this knowledge can aid in developing new antiviral strategies.

## Introduction

Pseudorabies virus (PRV) is a member of the family *Herpesviridae* that is related to herpes simplex virus 1 (HSV-1) (Mettenleiter, 2000; Klupp et al., 2004). PRV is the etiological agent of Aujeszky’s disease and can infect variety of mammals, including pigs, ruminants, carnivores, and rodents (Szpara et al., 2011). Interestingly, latent PRV infection can only occur in pigs, which are considered to be the natural host for this virus (Pomeranz et al., 2005; Fonseca et al., 2010). Aujeszky’s disease is characterized by abnormal nervous symptoms and death in newborn pigs, whereas older pigs exhibit respiratory disorders or reproductive failure (Sun et al., 2016). Since 2012, PRV variant virulent strains have been reported to be epidemic in China (Yu et al., 2014). PRV variants have been shown to be more virulent than the classical strains toward older pigs (Yang et al., 2016). Recently, PRV variants were reported to be related to acute human encephalitis cases (Liu et al., 2020).

For viral replication to occur in host cells, metabolites and energy are necessary for viral biopolymer synthesis and virion assembly. As low-molecular-weight compounds are the basis of metabolic activity, their levels can accurately reflect the response of host cells to viral infection (Munger et al., 2008; Amador-Noguez et al., 2010). Currently, improved metabolomics has made it possible to analyze the mechanisms through which viruses utilize low molecular weight metabolites in host cells. Metabolomics has shown great promise in uncovering complex virus–host interactions. For example, increased 7-dehydrocholesterol levels were shown to be associated with cholesterol metabolism disorder in host cells infected with hepatitis B virus (Rodgers et al., 2009). HSV-1 was shown to alter normal metabolic homeostasis in quiescent and growing cells and to stimulate aspects of glycolysis, the tricarboxylic acid(TCA) cycle, and pyrimidine biosynthetic metabolic pathways (Vastag et al., 2011). Although human cytomegalovirus (HCMV) belongs to the same family (*Herpesviridae*) as HSV-1, HCMV reorganizes its own metabolic program in host cells (Munger et al., 2006). Lipid metabolism is primarily regulated by HCMV to produce substrates for replication, whereas deoxypyrimidine metabolism is primarily regulated by HSV-1. Notably, the metabolic profiles of different host cell types infected with different HSV-1 strains are quite consistent, and the same phenomenon is observed in HCMV-infected cells (Munger et al., 2006; Vastag et al., 2011). This underlines the suggestion that reorganization of metabolic components might be the basic characteristic of virus replication in host cells, independent of the differences between multiple virus strains.

PRV has been demonstrated to hijack RNA transcription and protein synthesis to enhance its replication in host cells (Paulus et al., 2006; Yang et al., 2017). However, how PRV regulates the production of small molecule metabolites to promote macromolecular synthesis remains unclear. In this study, gas chromatography-mass spectrometry (GC-MS) was utilized to analyze the changes in the metabolic networks of PK-15 cells infected with different PRV strains, including the variant virulent GD-WH strain and the classical attenuated Bartha strain. Although the number of metabolites was different in PK-15 cells infected by the variant virulent strain and the classical attenuated strain at 8 or 16 h post infection (hpi), we found that most components altered in response to PRV infection belonged to consistent metabolic pathways such as glycolysis or amino acid and nucleotide metabolism. Further experiments showed that PRV replication was obviously down-regulated by the glucose depletion and glycolysis inhibitors including 2DG and oxamate. In addition, the prevention of oxidative phosphorylation in the TCA cycle had a minimal effect on viral replication, but the inhibition of the pentose phosphate pathway (PPP) also resulted in sharp declines of viral titers. Glutamine starvation resulted in the decline of viral titers, which could be restored by supplemental addition in the culture media. However, glutaminase activity inhibition or the supplement of 2-ketoglutarate into glutamine-deleted DMEM did not change the PRV replication in PK-15 cells. The results of the current study provide the first evidence that PRV reprograms the metabolic activity of PK-15 cells to benefit its infection. The variant virulent strain and the classical attenuated strain displayed similar metabolic features in PK-15 cells. These results may offer a better understanding of the biochemical materials utilized by PRV replication in host cells. This will be beneficial for exploring small molecular compounds that can be used as PRV replication inhibitors.

## Materials and Methods

### Cell Culture and Virus

The swine kidney cell line PK-15 (ATCC, CCL-33) was cultured in Dulbecco’s modified Eagle medium (DMEM) (11965, Gibico) supplemented with 10% fetal bovine serum (FBS) (Biological Industries, USA) at 37°C with 5% CO_2_. The classical attenuated strain (Bartha) was purchased from the China Veterinary Culture Collection Center (CVCC Number: AV249). The currently circulating variant virulent strain (GD-WH) (GenBank No. KT948051) was isolated from the brain of a pig suspected to be infected with PRV in 2015. Several rounds of plaque purification were conducted to obtain the pure virus. Viral titers were determined in PK-15 cells and were calculated as the 50% tissue culture infectious dose (TCID50) per milliliter according to the Spearman–Karber method (Reed and Muench, 1938).

### Growth Kinetics

A PK-15 cell monolayer cultured in a 25-cm^2^ cell culture flask was infected with the GD-WH or Bartha strain at a multiplicity of infection (MOI) of 10. At 4, 8, 12, 16, 20, 24, 28, 32, 36, and 40 hpi, 100 µl of cell supernatant was absorbed and stored at −80°C. The growth kinetics of each viral strain was analyzed according to viral titers in PK-15 cells.

### Sample Preparation for Metabolomics Assay

Approximately 2×10^6^ PK-15 cells were seeded into 6-cm dishes 24 h before viral infection. Each dish was marked according to preparation time, with four dishes prepared for each experimental group (24 total dishes). Cell monolayers were inoculated with the GD-WH or Bartha strain at an MOI of 10, with a monolayer inoculated with an equal volume of DMEM used as the mock infection. After 1 h, the inoculum was aspirated, and cells were washed twice with phosphate buffer saline (PBS). Subsequently, DMEM containing no FBS was added to each dish. All of the dishes were placed in an incubator for the indicated hours post infection.

At 8 or 16 hpi, the cell medium in each dish was discarded, and each dish was washed twice with cold PBS. Subsequently, the cells were quenched with 400 µl of cold methanol and chilled at −80°C for 30 min. Tridecanoic acid (91988, Sigma) and norvaline (53721, Sigma) premixed with methanol at a concentration of 1 μg/ml was used as a quality control (QC) and was maintained at -80°C. Cells were scraped from the dishes in 400 µl of ultrafiltered, cold water, and the cell mixture was used for subsequent analysis.

### Biochemical Intervention and Replenishment

For interfering with glycolysis, the TCA cycle, the PPP or glutamine metabolism, PK-15 cells with 80% confluence in 6-well cell culture plates were pretreated with one of the following inhibitors, e.g., 10 or 20 mM 2-deoxyglucose (2DG) (S4701, Selleck), 50 mM sodium oxamate, 1 μM oligomycin A, 500 μM 6-aminonicotinamide (6-AN), or 5 μM BPTES for 4 h. To accurately analyze the effects of biochemical intervention on virus replication, PK-15 cells were infected with the GD-WH strain at an MOI of 1. Then, cells were cultured in fresh medium containing corresponding inhibitors for 16 h. To analyze the effect of glucose starvation on PRV replication, PK-15 cells cultured in 6-well cell plates with 80% confluence were infected with PRV GD-WH strain at an MOI of 1. Then, cells were cultured in normal DMEM or depleted DMEM with no glucose, glutamine, and phenol red (A1443001, Gibico) supplemented with 2 mM L-glutamine (S1749, Selleck) for 16 h. To analyze the effect of glutamine starvation on PRV replication, PK-15 cells infected with PRV GD-WH strain at an MOI of 1 were cultured in normal DMEM or glutamine-free DMEM (11960077, Gibico) for 16 h. In the replenishment groups, different concentrations of L-glutamine (S1749, Selleck) or 2-ketoglutarate (S6237, Selleck) were added to the glutamine-free DMEM.

### Cell Viability Assay

A Cell Counting Kit-8 (CCK8) assay kit (C0037, Beyotime) was used to analyze the cell viability according to the manufacturer’s instructions. Briefly, PK-15 cells were cultured in 96-well culture plates at a seeding density of 1 × 10^4^ cells per well for 24 h. Then, the medium was replaced with 100 μl of DMEM containing one of the following inhibitors: 10 or 20 mM 2-deoxyglucose (2DG) (S4701, Selleck), 50 mM sodium oxamate, 1 μM oligomycin A, 500 μM 6-aminonicotinamide (6-AN), or 5 μM BPTES. After 16 h, cells were washed twice with PBS and cultured in 100 μl of DMEM containing 10 μl of CCK8 for 1 h at 37°C. The optical density was measured at 570 nm by using a model 680 microplate reader (Bio-Tek).

### Metabolite Extraction and Gas Chromatography-Mass Spectrometry Analysis

After the methanol content of the cell mixture was adjusted to 80%, cells were lysed by five rounds of ultra-sonication (a duration of 1 min) at 1 min intervals. After centrifugation at 14,000 g for 15 min at 4°C, 500 μl of each cell lysate supernatant was combined with 10 μl L-norleucine (50 μg/ml). Subsequently, the miscible liquid was evaporated to dryness under a stream of nitrogen. The residue was then redissolved in 30 μl of pyridine containing 20 mg/ml methoxyamine hydrochloride. Following an incubation at 37°C for 90 min, 30 μl of BSTFA (in 1% TMCS) was added. After the resulting mixture was derivatized at 70°C for 60 min, 2 μl of the mixture was analyzed by GC-MS (Agilent 7890A/5975C, Agilent Technologies). The QCs pooled from all samples were prepared and analyzed using the same procedure as that used for the experimental samples. The GC-MS analysis and data preprocessing methods were performed as described previously by Gou et al. (2017).

### Statistical Analysis of Gas Chromatography-Mass Spectrometry Data

For multivariate statistical analysis, including principal component analysis (PCA) and orthorhombic partial least-squares discriminant analysis (OPLS-DA), the data were normalized prior to being preprocessed by unit variance scaling and mean centering using SIMCA (version 14.1, Umetrics, Sweden). The R^2^X or R^2^Y and Q^2^ values were used to evaluate the model quality. R^2^X (PCA) or R^2^Y (PLS-DA and OPLS-DA) is described as the proportion of variance in the data explained by the model and indicates the goodness of fit, while Q^2^ is described as the proportion of variance in the data predicted by the model and indicates the predictability of the current model. To avoid model over-fitting, a default seven rounds of cross-validation in the SIMCA software was performed throughout the analysis.

### Identification and Statistical Analysis of Differential Metabolites

For univariate statistical analysis, the normalized data were analyzed in the “muma” software package in the R platform. A parametric test was performed on normally distributed data using Welch’s t-test, while a nonparametric test was performed on the non-normally distributed data using the Wilcoxon– Mann–Whitney test.

The variable importance in the projection (VIP) values (>1) in the OPLS-DA model and the *P* values (<0.05) from the univariate statistical analysis were used to identify potential differential metabolites. Fold change was calculated as the binary logarithm of the average normalized peak intensity ratio between Groups 1 and 2. A positive value indicates that the average mass response of Group 1 was higher than that of Group 2, whereas a negative value indicates a lower average mass response of Group 1 compared to Group 2.

## Results

### Growth Kinetics of the Variant Virulent and Classical Attenuated Pseudorabies Virus Strains

**Figure 1:**
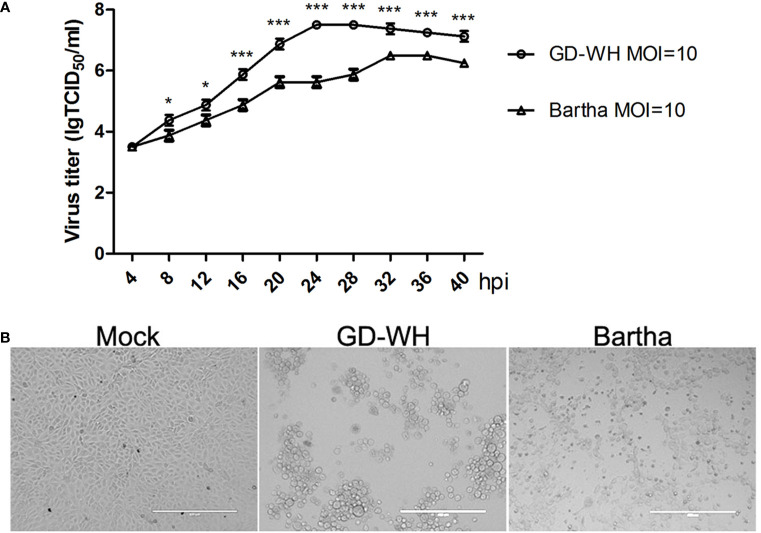
**(A)** Growth kinetics of the variant virulent and classical attenuated pseudorabies virus (PRV) strains. PK-15 cells were infected with the variant virulent (GD-WH) or classical attenuated (Bartha) PRV strains (MOI = 10), and the growth kinetics of each strain was analyzed as described in the Materials and Methods (mean ± SD; n=3; *P < 0.5; ^***^ *P* < 0.001). *P* values were calculated using two-way ANOVA. **(B)** Cytopathic effects of PK-15 cells infected with the variant virulent or classical attenuated PRV strains at 24 hpi. PK-15 cells were infected with the variant virulent (GD-WH) or classical attenuated (Bartha) PRV strains (MOI=10). At 24 hpi, the cytopathic effects of PRV infection of PK-15 cells were observed (scale bars=400 μm).

Viral titers of the variant virulent (GD-WH) strain were higher than that of the classical attenuated (Bartha) strain at each time point tested (**Figure 1A**). The peak viral titers of the GD-WH and Bartha strains were observed at 24 and 32 hpi, respectively. This result indicated that the GD-WH strain replicated faster than the Bartha strain in PK-15 cells. Both the GD-WH and Bartha PRV strains caused obvious cytopathologic effects in PK-15 cells at 24 hpi (**Figure 1B**). Considering that the GD-WH strain began to cause rounding and floating of almost all of the PK-15 cells by 24 hpi, PK-15 cells infected with the GD-WH and Bartha PRV strains at 8 and 16 hpi were sampled for subsequent GC-MS analysis to ensure that enough adherent cells were obtained.

### Principal Component Analysis of Metabolites Analyzed by Gas Chromatography-Mass Spectrometry

**Figure 2:**
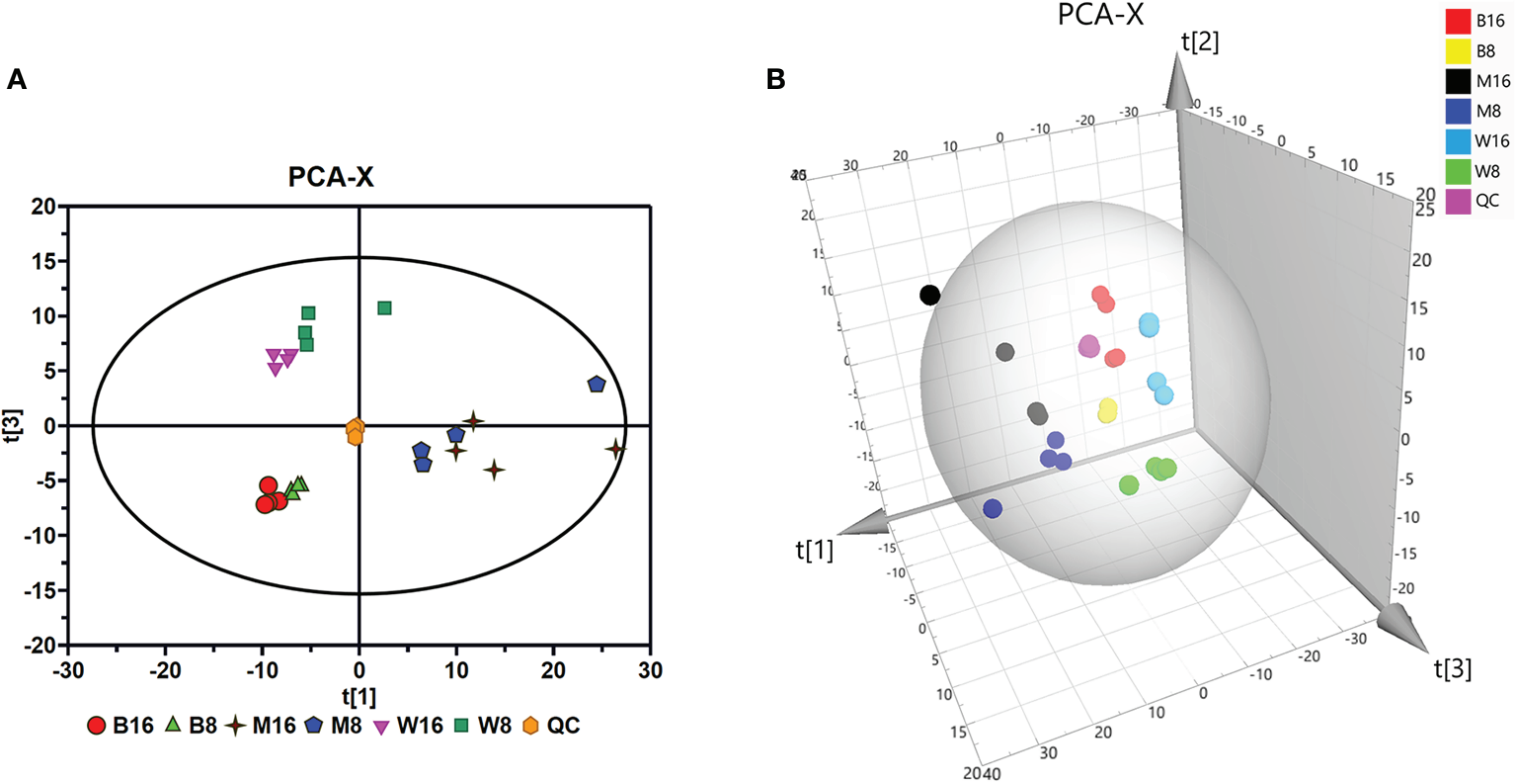
Principal component analysis (PCA) of gas chromatography-mass spectrometry (GC-MS) spectra. **(A)** Multivariate analysis of GC-MS spectra of metabolites using the PCA model. **(B)** Three-dimensional trajectory analysis of the PCA score plots. In all images, W8 and W16 represent the groups infected with the variant virulent (GD-WH) strain; B8 and B16 represent the groups infected with the classical attenuated (Bartha) strain; M8 and M16 represent the mock groups, and QC represents the quality control group.

The total ion current (TIC) chromatograms of all the samples in each group showed that the GC-MS method was suitable for the metabolite analyses performed in this study. Representative TIC chromatograms in each group are shown in **Figure S1**. The PCA model typically reflected the differences between the groups. To analyze whether PRV altered the composition of metabolites in PK-15 cells, the PCA model was used to analyze the data produced by the GC-MS analysis. The model cumulative interpretation rate, R^2^X (=0.809), demonstrated that PCA was suitable for determining the differences between the groups. In the PCA score map (**Figure 2A**), the QC group was concentrated in the middle region, demonstrating the good reliability and satisfactory reproducibility of the GC-MS method used in this study. The different distributions of the GD-WH and Bartha groups with mock groups at 8 and 16 hpi in the PCA score map indicated that PRV reprogrammed the metabolic composition of PK-15 cells. This result was further supported by different trajectory locations of the GD-WH and Bartha groups with mock groups in the three-dimensional score plots (**Figure 2B**).

### Differential Metabolite Analysis

**Figure 3:**
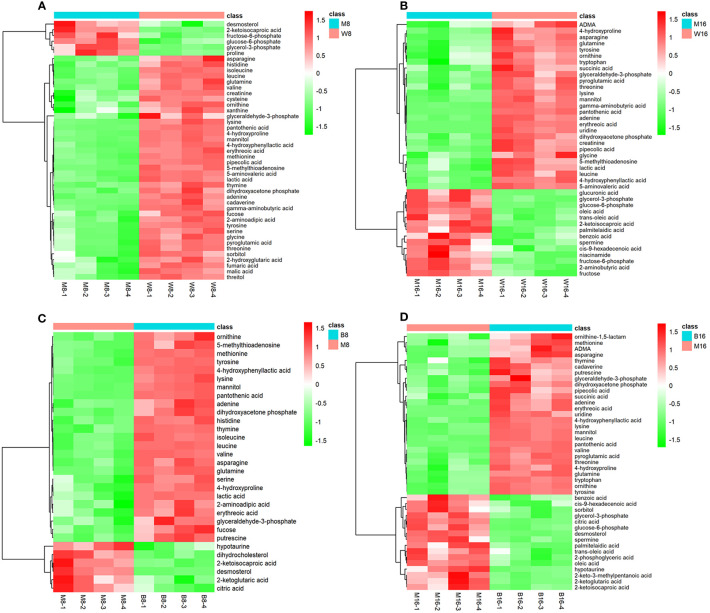
Heatmap visualization of differential metabolites in PK-15 cells infected with the variant virulent **(A, B)** or classical attenuated **(C, D)** PRV strains at 8 and 16 hpi. Rows: metabolites; columns: samples. The color from red (positive value) to green (negative value) of each rectangle was based on the ratio between pseudorabies virus (PRV)-infected groups vs mock groups. For example, a red color means that the average mass response of the metabolite in PRV-infected groups was greater than that in mock groups. In all images, W8 and W16 represent the groups infected with the variant virulent (GD-WH) strain; B8 and B16 represent the groups infected with the classical attenuated (Bartha) strain, and M8 and M16 represent the mock groups.

Because metabolomic analyses are more sensitive than transcriptomic or proteomics analyses, the OPLS-DA model was more successful in eliminating the effects of the genetic background and environmental disturbances compared with the PCA model. To precisely explore the changes in metabolites between the virus-infected and mock groups at different hpi, the OPLS-DA model was subsequently used to evaluate the GC-MS data. Similar to the PCA model, the OPLS-DA model also showed notably different distributions between the virus-infected and mock groups at each hpi. Furthermore, the results of the permutation test confirmed the good fit of this model (**Figure S2**). When the VIP value (>1) of the OPLS-DA model and the *P* value (<0.05) of the univariate statistical analysis were set, the differential metabolites were determined according to their fold changes. The numbers of metabolites altered in response to the GD-WH infection were 45 and 41 at 8 and 16 hpi (**Supplementary Tables S1** and **S2**, respectively). The numbers of differential metabolites in PK-15 cells infected with the Bartha strain were 31 and 43 at different hpi, shown in **Supplementary Tables S3** and **S4**, respectively. Heatmap analysis was performed to display the relationships of differential metabolites in PK-15 cells infected with the GD-WH or Bartha strain at 8 and 16 hpi. Our data indicated that 20 differential metabolites appeared on the heatmaps of both the GD-WH and Bartha strains at 8 hpi (**Figures 3A, C**); these included glyceraldehyde-3P, glycerone-P, lactate, 2-oxoglutarate, thymine, adenine, glutamine, and asparagine. Similarly, 19 differential metabolites appeared on the heatmaps of the GD-WH and Bartha strains at 16 hpi (**Figures 3B, D**). This tells us that different metabolic features may be associated with the GD-WH and Bartha strains infecting PK-15 cells.

### Metabolic Pathway Analysis

**Figure 4:**
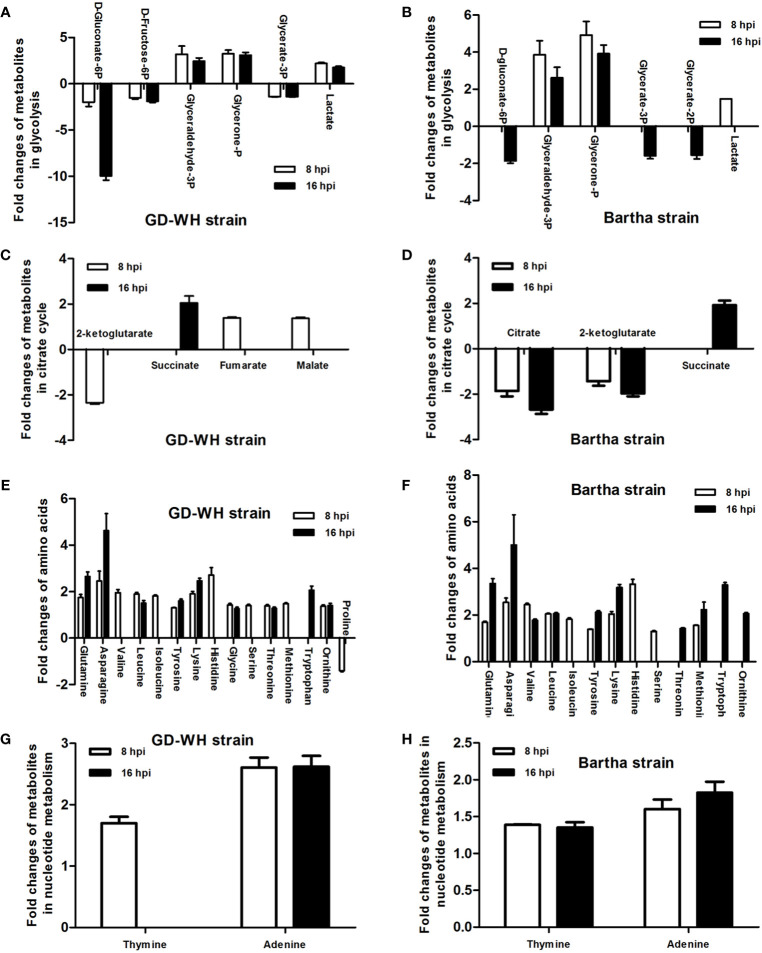
Statistical analysis of fold change of differential metabolites in PK-15 cells infected with the variant virulent **(A, C, E, G)** or classical attenuated **(B, D, F, H)** pseudorabies virus (PRV) strains at 8 and 16 hpi (mean ± SD; n=4). Fold change was calculated as a binary logarithm of the average mass response (normalized peak area) ratio between PRV-infected groups vs mock groups, where a positive value means that the average mass response of the metabolite in PRV-infected groups was greater than that in mock groups.

Changes in the levels of metabolites at crucial positions in networks are more likely to reflect the flow of pathways than changes at relatively marginal positions. To explore potential pathways in PK-15 cells altered in response to PRV infection, the connections of differential metabolites that appeared on the heatmaps of both the GD-WH and Bartha strains were identified with metabolic pathways according to the KEGG PATHWAY Database. As shown in **Figure 4**, many differential metabolites were related to glycolysis, TCA cycle, amino acid metabolism, and nucleotide metabolism at 8 and 16 hpi in the GD-WH or Bartha strains infecting PK-15 cells.

### Identification of the Effect of the Glycolysis on Pseudorabies Virus Replication

**Figure 5:**
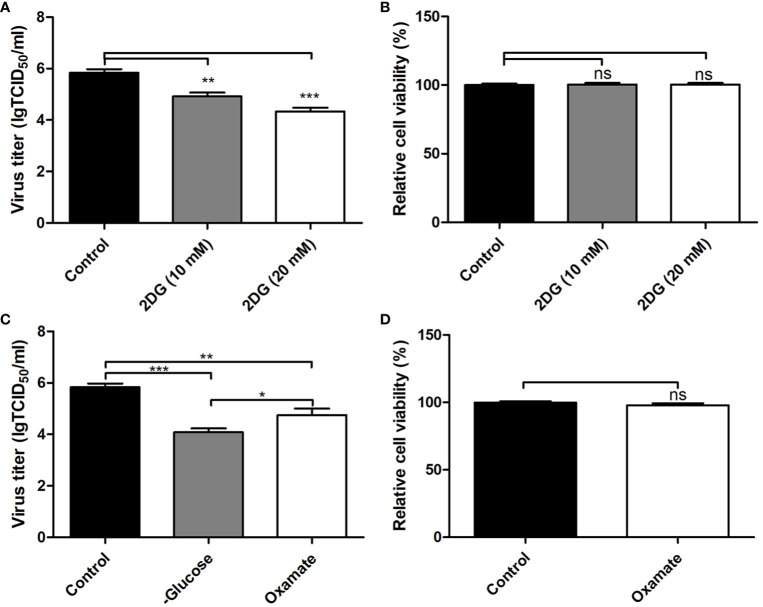
Inhibition of the glycolysis reduced the replication of pseudorabies virus (PRV) in PK-15 cells. **(A)** The effect of 2-deoxyglucose (2DG) treatment on the virus titers in PK-15 cells infected with the variant virulent PRV strain. PK-15 cells were pretreated with 10 or 20 mM 2DG for 3 h. Then, cells were infected with PRV GD-WH strain at an multiplicity of infection (MOI) of 1 and cultured in Dulbecco’s modified Eagle medium (DMEM) containing 10 or 20 mM 2DG. At 16 hpi, the titers of virus were analyzed as described in Materials and Methods (mean ± SD; n=3; ^**^ *P*<0.01; ^***^ *P* < 0.001). *P* values were calculated by using an unpaired Student’s t-test. **(B)** The effect of 2DG treatment on the cell viability of PK-15 cells. The cell viability of PK-15 cells treated with 10 and 20 mM 2DG was analyzed by the CCK8 assay as described in Materials and Methods (mean ± SD; n=3; *^NS^P*>0.05). **(C)** Glucose depletion or oxamate treatment decreased PRV titers in PK-15 cells. PK-15 cells were starved by be cultured in depletion DMEM repleted with 2 mM L-glutamine or pretreated with 50 mM oxamate for 3 h. Then cells were infected with PRV GD-WH strain at a MOI of 1. After being cultured in depletion DMEM repleted with 2 mM L-glutamine or DMEM containing 50 mM oxamate for 16 h, virus titers were analyzed as described in Materials and Methods (mean ± SD; n = 3; ^*^ *P* < 0.05; ^**^ *P* < 0.01; ^***^ *P* < 0.001). *P* values were calculated by using an unpaired Student’s t-test. **(D)** The effect of oxamate treatment on the cell viability of PK-15 cells. The cell viability of PK-15cells starved by glucose depletion or treated with oxamate for 16 h were analyzed by the CCK8 assay as described in Materials and Methods (mean ± SD; n = 3; ^NS^ *P* > 0.05).

The levels of gluconate-6-phosphate in PK-15 cells infected by the GD-WH strain was continuously down-regulated at 8 and 16 hpi, but decreased levels of gluconate-6-phosphate were only detected in PK-15 cells infected by the Bartha strain at 16 hpi (**Figures 4A, B**). In addition, decreased levels of D-fructose-6p were observed in glycolysis in PK-15 cells infected with the GD-WH strain at 8 and 16 hpi, but not in cells infected by the Bartha strain (**Figures 4A, B**). The different regulation levels of gluconate-6-phosphate and D-fructose-6p might be attributed to the slower replication speed of the Bartha strain than the GD-WH strain, and the basal content of these metabolites in PK-15 cells was sufficient for Bartha strain consumption at 8 hpi. Considering that glyceraldehyde-3P and glycerone-P were both increased in PK-15 cells infected by different PRV strains at 8 and 16 hpi (**Figures 4A, B**), we speculated that glycolysis might have been enhanced for PRV replication. To explore this hypothesis, we inhibited glycolysis of PRV-infected cells by using 2-deoxyglucose (2DG), a glucose analog that blocks glycolysis. PK-15 cells were infected with the GD-WH strain and then treated with different concentrations of 2DG (10 and 20 mM). The results indicated an obvious decrease of viral titers in 2DG-treated groups compared with the control groups, and 2DG treatment did not exhibit cellular cytotoxicity (**Figures 5A, B**). Next, the effect of glucose starvation on PRV replication was analyzed. The results showed that viral titers were clearly decreased in PK-15 cells cultured in DMEM lacking glucose (**Figure 5C**). Notably, a tendency toward increased lactate levels was observed in PK-15 cells infected by the GD-WH and Bartha strains at 8 hpi, but the level of lactate was slightly up-regulated in PK-15 cells infected by the GD-WH strain at 16 hpi (**Figures 4A, B**). This indicated that the fold change of lactate content in PRV-infected cells was higher at 8 than 16 hpi, which was not consistent with the time-dependent decreased levels of gluconate-6-phosphate. Therefore, we speculated that the gluconate-6-phosphate in the glycolysis pathway might be converted to other pathways during PRV infection, for example the TCA cycle or the PPP, in addition to being responsible for the production of lactate. When the lactate production was prevented by inhibiting the lactate dehydrogenase (LDH) enzyme using oxamate, a smaller decline of viral titers was observed than that in the glucose depletion or 2DG treatment PK-15 cells (**Figure 5C**). In addition, oxamate did not reduce the cell viability by 16 h (**Figure 5D**). Because lactate is in the downstream segment of glycolysis, the smaller inhibitory effect of oxamate on viral replication than glucose depletion supported our speculation that glycolysis is linked to the TCA cycle or the PPP during PRV infection of PK-15 cells.

### The Effect of the Tricarboxylic Acid Cycle and the Pentose Phosphate Pathway on Pseudorabies Virus Replication

**Figure 6:**
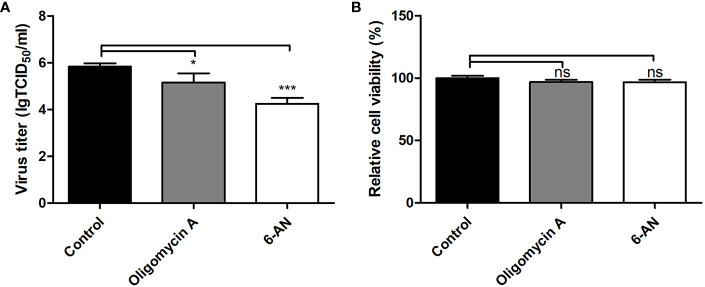
The effect of the TCA cycle and pentose phosphate pathway (PPP) on pseudorabies virus (PRV) replication in PK-15 cells. **(A)** The effect of oligomycin A or 6-AN treatment on PRV replication. PK-15 cells were pretreated with 1 μM Oligomycin A or 500 μM 6-AN for 3 h. Then cells were infected with PRV GD-WH strain at a MOI of 1 and cultured in DMEM containing 1 μM Oligomycin A or 500 μM 6-AN. At 16 hpi, the titers of virus were analyzed as described in Materials and Methods (mean ± SD; n = 3; ^*^ *P* < 0.05; ^***^ *P* < 0.001). *P* values were calculated by using an unpaired Student’s t-test. **(B)** The effect of Oligomycin A or 6-AN treatment on the cell viability of PK-15 cells. The cell viability of PK-15 cells treated with 1 μM Oligomycin A or 500 μM 6-AN were analyzed by the CCK8 assay as described in Materials and Methods (mean ± SD; n = 3; *^NS^P* > 0.05).

In the TCA cycle, only a few metabolites displayed relatively small changes in PK-15 cells infected by the GD-WH and Bartha strains. Citrate showed a declining tendency in PK-15 cells infected by the Bartha strain at 8 and 16 hpi, but not when infected by the GD-WH strain. However, fumarate and malate in the TCA cycle were only increased in PK-15 cells infected by the GD-WH strain at 8 hpi. Decreased levels of 2-ketoglutarate were detected in PK-15 cells infected by different strains of PRV at 8 hpi, but levels of succinate were increased by PRV infection at 16 hpi (**Figures 4C, D**). Given the minimal and independent changes of these metabolites in the TCA cycle, we speculate that the pathway responsible for oxidative phosphorylation and ATP production might not be entirely up-regulated by PRV infection. Possibly only several metabolites were used as carbon sources. To analyze the role of the TCA cycle in PRV replication, oligomycin A was used to prevent oxidative phosphorylation in PK-15 cells infected by PRV. We only observed a minimal decline of viral titer (**Figure 6A**). This indicated that oxidative phosphorylation depending on the TCA cycle is not the key factor for virion production during PRV infection. Meanwhile, it excludes the possibility that the inhibition of glycolysis by the absence of glucose or 2DG reduced PRV replication *via* indirectly affecting the TCA cycle. Furthermore, the PPP in PK-15 cells was inactivated by using 6-aminonicotinamide (6AN), an inhibitor of the PPP enzyme glucose-6-dehydrogenase. The result showed that 6AN had a sharply inhibitory effect on viral titers that was the same as in the glucose starvation and 2DG treatments (**Figure 6A**). This was consistent with our speculation that gluconate-6-phosphate enters the PPP to enhance the viral nucleotide synthesis during PRV infection. Also, neither oligomycin A nor 6-AN treatment reduced the cell viability during 16 h (**Figure 6B**).

### Glutamine Is an Essential Factor for Pseudorabies Virus Replication

**Figure 7:**
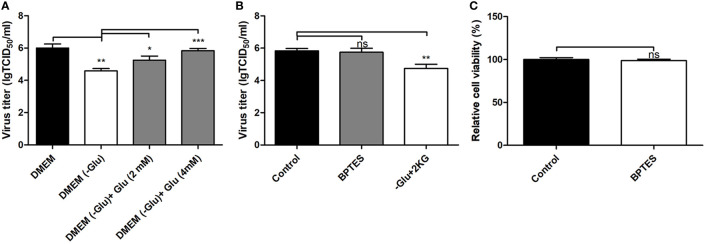
Depletion of glutamine reduced pseudorabies virus (PRV) replication in PK-15 cells in a manner independent of the TCA cycle. **(A)** Glutamine starvation had a repressive effect on PRV replication in PK-15 cells. PK-15 cells were starved by being cultured in glutamine-free DMEM for 3 h. Then, cells were infected with PRV GD-WH strain at an multiplicity of infection (MOI) of 1 and cultured in normal Dulbecco’s modified Eagle medium (DMEM) or no glutamine DMEM. In replenishment groups, 2 or 4 mM L-glutamine was added to glutamine-free DMEM. At 16 h, the titers of virus were analyzed as described in Materials and Methods (mean ± SD; n=3; ^*^ *P*<0.05; ^**^ *P*<0.01; ^***^ *P* < 0.001). *P* values were calculated by using an unpaired Student’s t-test. **(B)** BPTES treatment had no effect on PRV replication in PK-15 cells. PK-15 cells were pretreated with 1 μM BPTES for 3 h. Then cells were infected with PRV GD-WH strain at a MOI of 1 and cultured in DMEM containing 1 μM BPTES. At 16 hpi, the titers of virus were analyzed as described in Materials and Methods (mean ± SD; n = 3; *^NS^P* > 0.05; **P < 0.01). *P* values were calculated by using an unpaired Student’s t-test. **(C)** The effect of BPTES treatment on the cell viability of PK-15 cells. The cell viability of PK-15cells treated with 1 μM BPTES were analyzed by the CCK8 assay as described in Materials and Methods (mean ± SD; n = 3; *^NS^P* > 0.05). **(C)** 2-ketoglutarate supplement cannot recover the effect of glutamine starvation on PRV replication in PK-15 cells. PK-15 cells were starved by being cultured in no glutamine DMEM for 3 h. Then cells were infected with PRV GD-WH strain at a MOI of 1 and cultured in normal DMEM or no glutamine DMEM. In 2-ketoglutarate supplement groups, 5 mM 2-ketoglutarate was added in no glutamine DMEM. At 16 h, the titers of virus were analyzed as described in Materials and Methods (mean ± SD; n = 3; *^NS^P* > 0.05). *P* values were calculated by using an unpaired Student’s t-test.

The levels of multiple amino acids were changed in PK-15 cells infected by the different PRV strains at 8 and 16 hpi. These included glutamine, asparagine, histidine, leucine, and lysine. These amino acids might be related to protein synthesis or to other metabolic pathways. It has been reported that glutamine can be employed as a material for nucleic acid synthesis during HSV-1 infection (Vastag et al., 2011). Because PRV is a DNA virus with a large genome as in HSV-1, this study focused on the function of glutamine on PRV replication. By cultivating the GD-WH strain with glutamine-deleted DMEM, we found that deletion of glutamine caused a significant reduction of infectious viral particles at 16 hpi. However, viral titers increased in a dose-dependent manner after 2 or 4 mM glutamine was supplemented into glutamine-deleted DMEM (**Figure 7A**). These data suggested that glutamine is essential for PRV replication in PK-15 cells. In addition to being used as a material for nucleotide biosynthesis, it is known that glutamine can also be converted to glutamate by the enzymatic activity of glutaminase (GLS). Subsequently, glutamate can enter the TCA cycle *via* conversion to 2-ketoglutarate by glutamate dehydrogenase (GDH). Hence, we further explored whether PRV utilized glutamine to supply the TCA cycle. When the GLS activity was interfered by using the BPTES in PK-15 cells, PRV replication was unchanged (**Figure 7B**). Moreover, the supplement of 2-ketoglutarate into glutamine-deleted DMEM did not prevent the decline of viral replication in PK-15 cells infected by PRV (**Figure 7B**). In addition, we demonstrated that BPTES is not cytopathic to the cell viability (**Figure 7C**). These results suggested that glutamine was mainly connected to nucleotide biosynthesis in PK-15 cells infected by PRV and was not converted to 2-ketoglutarate in the TCA cycle.

## Discussion

**Figure 8:**
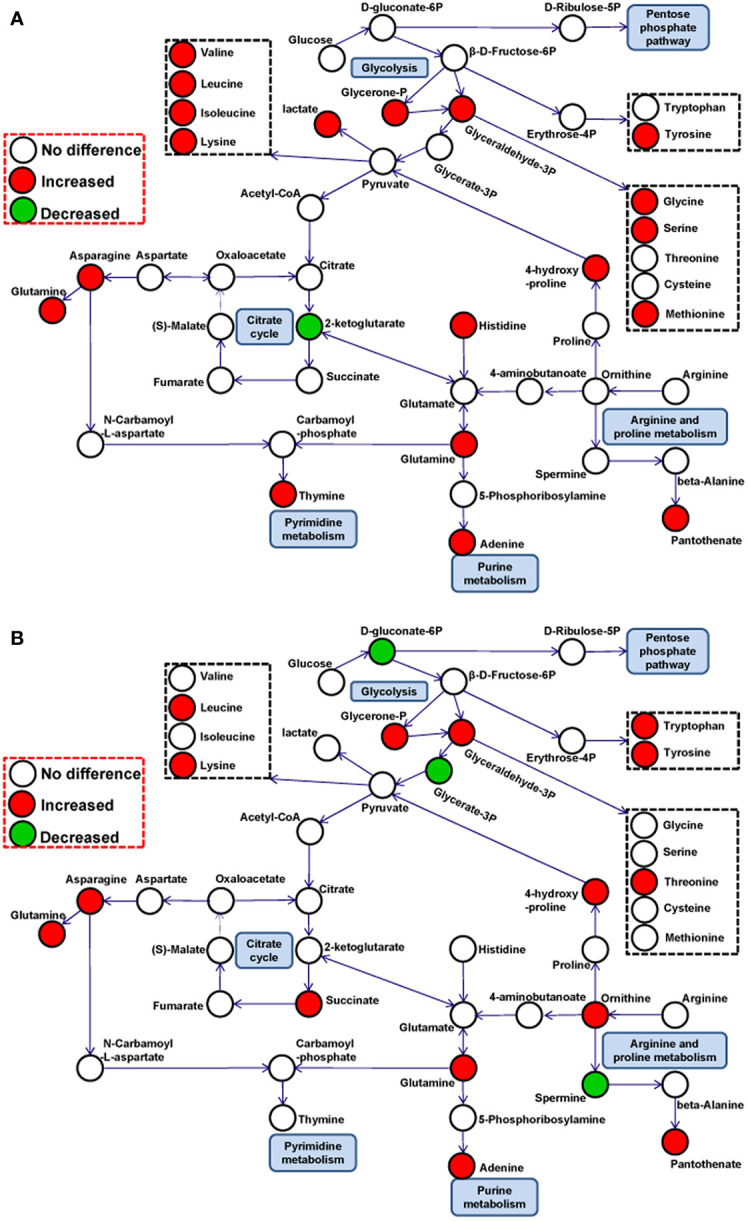
Schematic overview of metabolic pathways in PK-15 cells infected by different pseudorabies virus (PRV) strains at 8 **(A)** and 16 **(B)** hpi. The metabolites are shown in different colors according to their changes: red indicates increased metabolites; green indicates decreased metabolites, and white indicates no difference metabolites.

The development of metabolomics makes it possible to perform systematic analyses of complicated cellular metabolic networks, thus offering a new perspective for understanding the basic replication characteristics of viruses in their host cells (Munger et al., 2008). To explore how PRV reprograms metabolic features to benefit its replication in host cells, PK-15 cells were selected to be a viral infection model, a method that is typically used to research PRV growth *in vitro* (Yu et al., 2016). To reveal the basic metabolic characteristics in PRV-infected cells, the variant virulent GD-WH and classical attenuated Bartha strains were used to infect PK-15 cells, and the cells were analyzed by GC-MS in this study. The virulent variant strain was reported to be epidemic in China since 2012, and the Bartha strain was an attenuated vaccine that has been widely administered in the global swine industry since the 1970s (Yu et al., 2014). The GC-MS data showed that many metabolites were increased in glycolysis, the TCA cycle, amino acid metabolism, and nucleic acid metabolism in PK-15 cells infected by the different viral strains. The common metabolic profile of PK-15 cells infected by these different PRV strains demonstrated that metabolite reorganization might be a fundamental biochemical requirement for viral replication, which has also been reported in HSV-1 or HCMV-infected cells (Vastag et al., 2011). Interestingly, although PRV is closely associated with HSV-1, many amino acids in PRV-infected PK-15 cells displayed a reverse tendency compared with HSV-1 infected host cells. We speculate that different viruses might organize their own metabolic networks to serve viral replication. By establishing the relationships between potential pathways, the metabolic networks in PK-15 cells infected by PRV at 8 (**Figure 8A**) and 16 hpi (**Figure 8B**) could be predicted.

In mammalian cells, glycolysis and the TCA cycle are key processes in central carbon metabolism, linking glucose to energy production and amino acid, lipid, and nucleotide biosynthesis. In glycolysis, the levels of glycerol-3-phosphate and glycerone-P were increased at 8 (**Figure 8A**) and 16 (**Figure 8B**) hpi for both viral strains. This indicated that PRV activated the glycolysis pathway in PK-15 cells during the infection stage. The time-dependent consumption of gluconate-6-phosphate in PRV-infected cells further supported this hypothesis. It has been reported that the glycolysis pathway is enhanced by some viruses to benefit their replication. For example, the replication of HSV-1 and norovirus rely on active glycolysis in host cells (Abrantes et al., 2012; Passalacqua et al., 2019). Our results showed that replication of PRV in PK-15 cells could be obviously down-regulated by glucose depletion and the glycolysis pathway inhibitor 2DG or oxamate. However, we only observed a minimal decline of viral titer after oxidative phosphorylation in PK-15 cells was interfered with by using the oligomycin A. Furthermore, we found that the inhibition of the PPP had a clearly inhibitory effect on viral titers in PRV-infected PK-15 cells. These results indicated that glycolysis and PPP is mainly promoted and utilized for PRV replication in PK-15 cells, but not the oxidative phosphorylation in the TCA cycle. It has been shown that the TCA cycle is modulated to promote pyrimidine production by HSV-1 or for fatty acid biosynthesis by HCMV (Munger et al., 2006; Vastag et al., 2011). Considering that citrate and 2-ketoglutarate can be converted to succinate, the opposite change trends of 2-ketoglutarate and succinate in PRV-infected PK-15 cells support the suggestion that succinate in the TCA cycle might be employed as a central carbon source that is subsequently connected with amino acids or nucleotide metabolism but is not involved in ATP production (**Figures 8A, B**). Although PRV belongs to the same family as HSV-1 and HCMV, the flow of the enhanced central carbon metabolism in PK-15 cells needs to be further elucidated in future studies.

Previous reports showed that amino acid metabolism was disturbed during dengue virus infection and that amino acids may be converted into other biomolecules such as pyruvate and acetyl-coA (Birungi et al., 2010). The results of previous proteomic analyses showed that 21 proteins were up-regulated in the protein processing pathway in the endoplasmic reticulum of PK-15 cells infected by PRV (Yang et al., 2017). Whether the increased levels of amino acids observed in this study were associated with enhanced protein synthesis needs to be further explored. It has been demonstrated that glutamine is a key factor for the replication of some viruses (Vastag et al., 2011; Fontaine et al., 2014). Considering this, the role of glutamine in PRV replication was explored in the present study. We found that viral titers of PRV were decreased in the absence of glutamine in the culture medium. Further experiments showed that glutamine supplementation could rescue the replication of PRV. In addition to glucose, glutamine is a primary carbon donor for the TCA cycle. It has been shown that HCMV and vaccinia virus can manipulate glutamine metabolism to compensate for the diversion of glucose *via* the TCA cycle (Chambers et al., 2010; Fontaine et al., 2014). However, inhibition of GLS activity or supplementing 2-ketoglutarate into glutamine-deleted DMEM did not affect PRV replication in PK-15 cells. This indicates that glutamine did not enter the TCA cycle in PRV-infected PK-15 cells, in contrast to host cells infected by HCMV or vaccinia virus. We speculate that PRV utilizes glutamine to serve in purine or thymine synthesis, as has been reported in host cells infected by HSV-1 or gallid alpha herpesvirus 1 (Qiao et al., 2020).

The adenine and thymine contents in PK-15 cells were increased by both the GD-WH and Bartha PRV strain at 8 hpi (**Figures 4G, H**). These results are consistent with previous reports, where the levels of deoxyadenosine triphosphate (dTMP) and deoxythymidine triphosphate (dTTP) were shown to continuously increase in growth-arrested fibroblasts throughout HSV-1 infection (Vastag et al., 2011). Like HSV-1, PRV is a double-stranded DNA virus with an approximately 140-kb genome. Hence, it is essential for PRV to manipulate nucleotide precursors in host cells for viral DNA synthesis. In addition, our study showed that uridine levels increased in PK-15 cells during infection by the GD-WH and Bartha strains; this can occur in DNA as a result of cytosine deamination or through the misincorporation of dUTP (Chen et al., 2002). It has been shown that dUTPase (UL50) from HSV-1 can reduce the incorporation of uracil into genomic DNA by increasing the conversion of dUTP to dUMP (Bogani et al., 2009). Furthermore, uracil-DNA glycosylase (UL2) is responsible for the accurate removal of uracil from viral DNA (Bogani et al., 2010). Further studies are needed to ascertain whether the extra uridine content in PK-15 cells infected by the GD-WH or Bartha strain was attributed to the presence of similar viral enzymes as in HSV-1.

In this study, we generated the first metabolic profiles of PK-15 cells infected by the variant virulent or classical attenuated PRV strains. PRV infection primarily affected the metabolic pathways in PK-15 cells, including glycolysis, amino acid metabolism, and nucleotide metabolism, but there was minimal regulation of the TCA cycle. Interestingly, the metabolic profiles were similar for the variant virulent and classical attenuated strains during infection of PK-15 cells. Although PRV belongs to the same family as HSV-1 and HCMV, it leads to different metabolic consequences. The results of this study may clarify the biochemical materials utilized by PRV replication in host cells.

## Data Availability Statement

The original contributions presented in the study are included in the article/**Supplementary Material**; further inquiries can be directed to the corresponding author.

## Author Contributions

HG carried out the data analysis and drafted the manuscript. ZB, ZJ, and PC participated in the experiments. SS, YL, and KZ participated in the data analysis. RC and CL conceived of the study. DY prepared the materials for the experiments. All authors contributed to the article and approved the submitted version.

## Fuinding

This work was supported by grants from the Key Projects in the National Research and Development Program during the thirteenth Five-year Plan Period (No. 2018YFD0500804), the Natural Science Foundation of China (No. 31902273), the Natural Science Foundation of Guangdong, China (Nos. 2019A1515010757 and 2020A1515010475), the Key Areas of Research and Development Program of Guangdong, China (No. 2019B020217002), the Science and Technology plan Program of Guangdong, China (No. 2017B020202002), the Science and Technology Program of Guangzhou, China (No. 201804010071), the 2018 Rural Revitalization Strategy Project (No. Guangdong Agriculture Planning 2018-54), and the Special Fund for Scientific Innovation Strategy-Construction of High Level Academy of Agriculture Science (No. R2017YJ-YB2005 and R2018QD-094).

## Conflict of Interest

The authors declare that the research was conducted in the absence of any commercial or financial relationships that could be construed as a potential conflict of interest.

## References

[B1] Abrantes J. L. Alves C. M. Costa J. Almeida F. C. Sola-Penna M. Fontes C. F. (2012). Herpes simplex type 1 activates glycolysis through engagement of the enzyme 6-phosphofructo-1-kinase (PFK-1). Biochim. Biophys. Acta 1822 (8), 1198–1206. 10.1016/j.bbadis.2012.04.011 22542512

[B2] Amador-Noguez D. Feng X. J. Fan J. Roquet N. Rabitz H. Rabinowitz J. D. (2010). Systems-level metabolic flux profiling elucidates a complete, bifurcated tricarboxylic acid cycle in Clostridium acetobutylicum. J. Bacteriol. 192 (17), 4452–4461. 10.1128/JB.00490-10 20622067 PMC2937365

[B3] Birungi G. Chen S. M. Loy B. P. Ng M. L. Li S. F. (2010). Metabolomics approach for investigation of effects of dengue virus infection using the EA.hy926 cell line. J. Proteome Res. 9 (12), 6523–6534. 10.1021/pr100727m 20954703

[B4] Bogani F. Chua C. N. Boehmer P. E. (2009). Reconstitution of uracil DNA glycosylase-initiated base excision repair in herpes simplex virus-1. J. Biol. Chem. 284 (25), 16784–16790. 10.1074/jbc.M109.010413 19411250 PMC2719314

[B5] Bogani F. Corredeira I. Fernandez V. Sattler U. Rutvisuttinunt W. Defais M. (2010). Association between the herpes simplex virus-1 DNA polymerase and uracil DNA glycosylase. J. Biol. Chem. 285 (36), 27664–27672. 10.1074/jbc.M110.131235 20601642 PMC2934634

[B6] Chambers J. W. Maguire T. G. Alwine J. C. (2010). Glutamine metabolism is essential for human cytomegalovirus infection. J. Virol. 84 (4), 1867–1873. 10.1128/JVI.02123-09 19939921 PMC2812398

[B7] Chen R. Wang H. Mansky L. M. (2002). Roles of uracil-DNA glycosylase and dUTPase in virus replication. J. Gen. Virol. 83 (Pt 10), 2339–2345. 10.1099/0022-1317-83-10-2339 12237414

[B8] Fonseca A. A. Jr. Camargos M. F. de Oliveira A. M. Ciacci-Zanella J. R. Patricio M. A. Braga A. C. (2010). Molecular epidemiology of Brazilian pseudorabies viral isolates. Vet. Microbiol. 141 (3-4), 238–245. 10.1016/j.vetmic.2009.09.018 19828266

[B9] Fontaine K. A. Camarda R. Lagunoff M. (2014). Vaccinia virus requires glutamine but not glucose for efficient replication. J. Virol. 88 (8), 4366–4374. 10.1128/JVI.03134-13 24501408 PMC3993723

[B10] Gou H. Zhao M. Yuan J. Xu H. Ding H. Chen J. (2017). Metabolic Profiles in Cell Lines Infected with Classical Swine Fever Virus. Front. Microbiol. 8:691:691. 10.3389/fmicb.2017.00691 28473819 PMC5397473

[B11] Klupp B. G. Hengartner C. J. Mettenleiter T. C. Enquist L. W. (2004). Complete, annotated sequence of the pseudorabies virus genome. J. Virol. 78 (1), 424–440. 10.1128/jvi.78.1.424-440.2004 14671123 PMC303424

[B12] Liu Q. Wang X. Xie C. Ding S. Yang H. Guo S. (2020). A novel human acute encephalitis caused by pseudorabies virus variant strain. Clin. Infect. Dis. ciaa987. 10.1093/cid/ciaa987 32667972

[B13] Mettenleiter T. C. (2000). Aujeszky’s disease (pseudorabies) virus: the virus and molecular pathogenesis–state of the art, June 1999. Vet. Res. 31 (1), 99–115. 10.1051/vetres:2000110 10726640

[B14] Munger J. Bajad S. U. Coller H. A. Shenk T. Rabinowitz J. D. (2006). Dynamics of the cellular metabolome during human cytomegalovirus infection. PloS Pathog. 2 (12), e132. 10.1371/journal.ppat.0020132 17173481 PMC1698944

[B15] Munger J. Bennett B. D. Parikh A. Feng X. J. McArdle J. Rabitz H. A. (2008). Systems-level metabolic flux profiling identifies fatty acid synthesis as a target for antiviral therapy. Nat. Biotechnol. 26 (10), 1179–1186. 10.1038/nbt.1500 18820684 PMC2825756

[B16] Passalacqua K. D. Lu J. Goodfellow I. Kolawole A. O. Arche J. R. Maddox R. J. (2019). Glycolysis Is an Intrinsic Factor for Optimal Replication of a Norovirus. mBio 10 (2), e02175-18. 10.1128/mBio.02175-18 30862747 PMC6414699

[B17] Paulus C. Sollars P. J. Pickard G. E. Enquist L. W. (2006). Transcriptome signature of virulent and attenuated pseudorabies virus-infected rodent brain. J. Virol. 80 (4), 1773–1786. 10.1128/JVI.80.4.1773-1786.2006 16439534 PMC1367157

[B18] Pomeranz L. E. Reynolds A. E. Hengartner C. J. (2005). Molecular biology of pseudorabies virus: impact on neurovirology and veterinary medicine. Microbiol. Mol. Biol. Rev. 69 (3), 462–500. 10.1128/MMBR.69.3.462-500.2005 16148307 PMC1197806

[B19] Qiao Y. Wang Z. Han Z. Shao Y. Ma Y. Liang Y. (2020). Global exploration of the metabolic requirements of gallid alphaherpesvirus 1. PloS Pathog. 16 (8), e1008815. 10.1371/journal.ppat.1008815 32833996 PMC7470321

[B20] Reed L. J. Muench H. (1938). A SIMPLE METHOD OF ESTIMATING FIFTY PER CENT ENDPOINTS12. Am. J. Epidemiol. 27 (3), 493–497. 10.1093/oxfordjournals.aje.a118408

[B21] Rodgers M. A. Saghatelian A. Yang P. L. (2009). Identification of an overabundant cholesterol precursor in hepatitis B virus replicating cells by untargeted lipid metabolite profiling. J. Am. Chem. Soc. 131 (14), 5030–5031. 10.1021/ja809949r 19301856 PMC4166558

[B22] Sun Y. Luo Y. Wang C. H. Yuan J. Li N. Song K. (2016). Control of swine pseudorabies in China: Opportunities and limitations. Vet. Microbiol. 183, 119–124. 10.1016/j.vetmic.2015.12.008 26790944

[B23] Szpara M. L. Tafuri Y. R. Parsons L. Shamim S. R. Verstrepen K. J. Legendre M. (2011). A wide extent of inter-strain diversity in virulent and vaccine strains of alphaherpesviruses. PloS Pathog. 7 (10), e1002282. 10.1371/journal.ppat.1002282 22022263 PMC3192842

[B24] Vastag L. Koyuncu E. Grady S. L. Shenk T. E. Rabinowitz J. D. (2011). Divergent effects of human cytomegalovirus and herpes simplex virus-1 on cellular metabolism. PloS Pathog. 7 (7), e1002124. 10.1371/journal.ppat.1002124 21779165 PMC3136460

[B25] Yang Q. Y. Sun Z. Tan F. F. Guo L. H. Wang Y. Z. Wang J. (2016). Pathogenicity of a currently circulating Chinese variant pseudorabies virus in pigs. World J. Virol. 5 (1), 23–30. 10.5501/wjv.v5.i1.23 26870671 PMC4735551

[B26] Yang S. Pei Y. Zhao A. (2017). iTRAQ-based Proteomic Analysis of Porcine Kidney Epithelial PK15 cells Infected with Pseudorabies virus. Sci. Rep. 7, 45922. 10.1038/srep45922 28374783 PMC5379687

[B28] Yu X. Zhou Z. Hu D. Zhang Q. Han T. Li X. (2014). Pathogenic pseudorabies virus, Chin. Emerg. Infect. Dis. 20 (1), 102–104. 10.3201/eid2001.130531 24377462 PMC3884716

[B27] Yu T. Chen F. Ku X. Fan J. Zhu Y. Ma H. (2016). Growth characteristics and complete genomic sequence analysis of a novel pseudorabies virus in China. Virus Genes 52 (4), 474–483. 10.1007/s11262-016-1324-z 27012685

