# Supplementary material for: Metabolomics Exploration of Pseudorabies Virus Reprogramming Metabolic Profiles of PK-15 Cells to Enhance Viral Replication
Source: Front Cell Infect Microbiol. 2021 Jan 29;10:599087. doi: 10.3389/fcimb.2020.599087 (PMC7879706; doi:10.3389/fcimb.2020.599087)
Supplement: Supplementary file 1 [file DataSheet_1.docx]

1 Supplementary Figures and Tables

1.1 Supplementary Figures


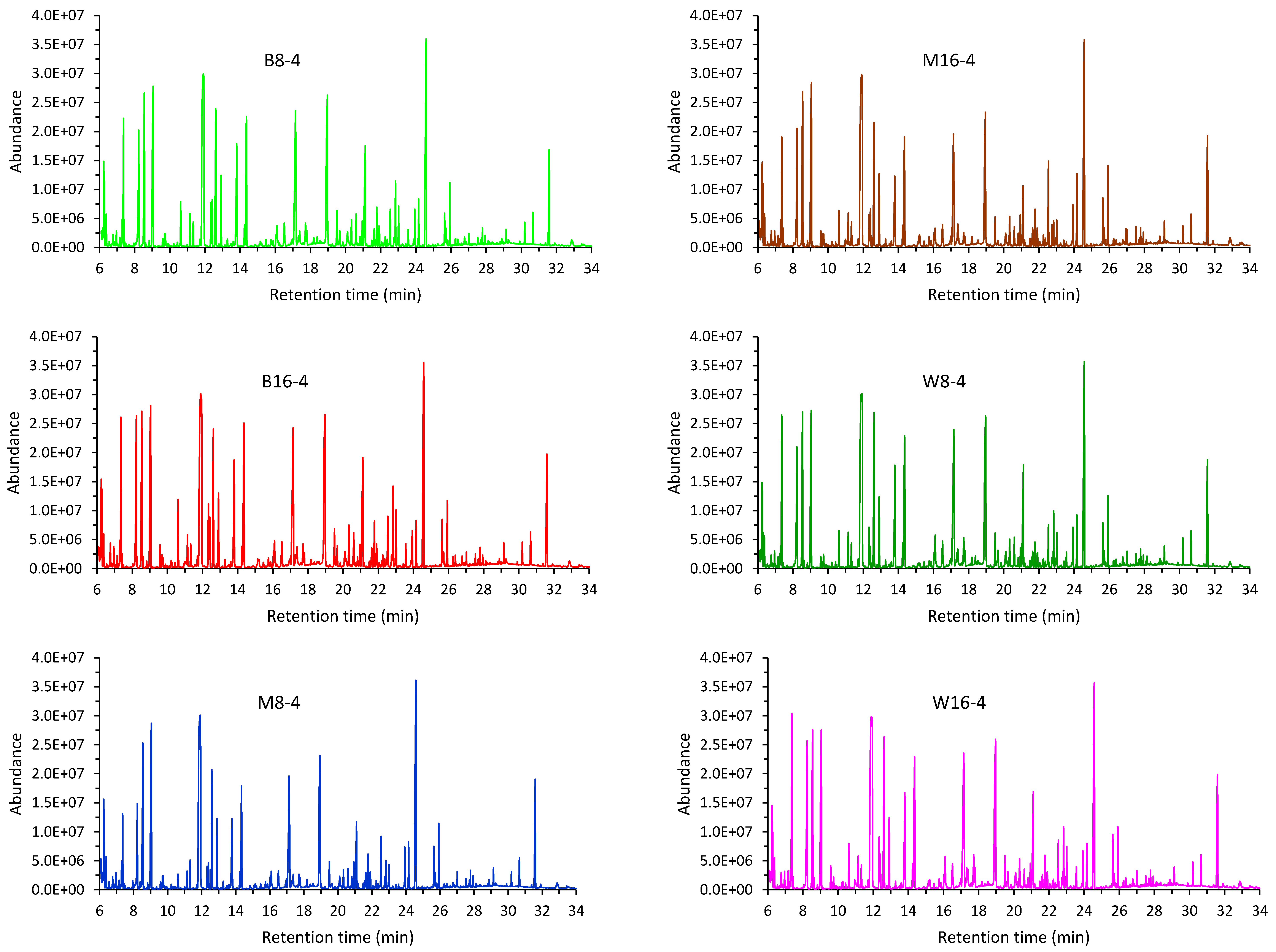


**Figure S1.** Typical total ion chromatograms of metabolites extracted from PK-15 cells. PK-15 cells were infected with the virulent variant (GD-WH) or classical attenuated (Bartha) strain PRV (MOI=10). At 8 and 16 hpi, metabolites were respectively extracted and analyzed by the GC-MS method. In all images, W8 and W16 represented groups infected with the virulent variant (GD-WH) strain, B8 and B16 represented groups infected with the classical attenuated (Bartha) strain, M8 and M16 represented mock groups.


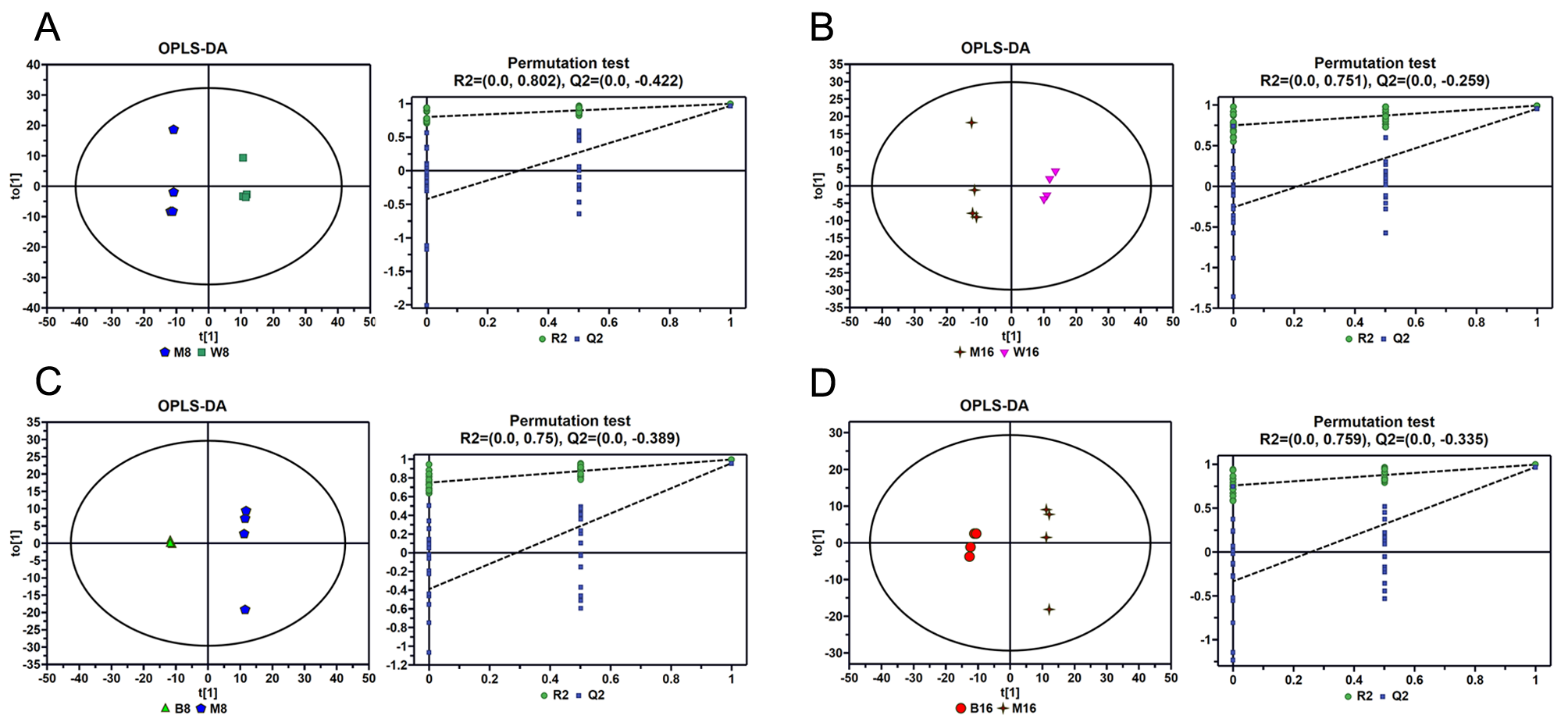


**Figure S2.** OPLS-DA of GC-MS spectra. (A, B, C and D) Multivariate statistical analysis of GC-MS spectra of metabolites by the OPLS-DA model. The permutation test confirmed the good fit of this model. In all images, W8 and W16 represented groups infected with the virulent variant (GD-WH) strain, B8 and B16 represented groups infected with the classical attenuated (Bartha) strain, M8 and M16 represented mock groups.


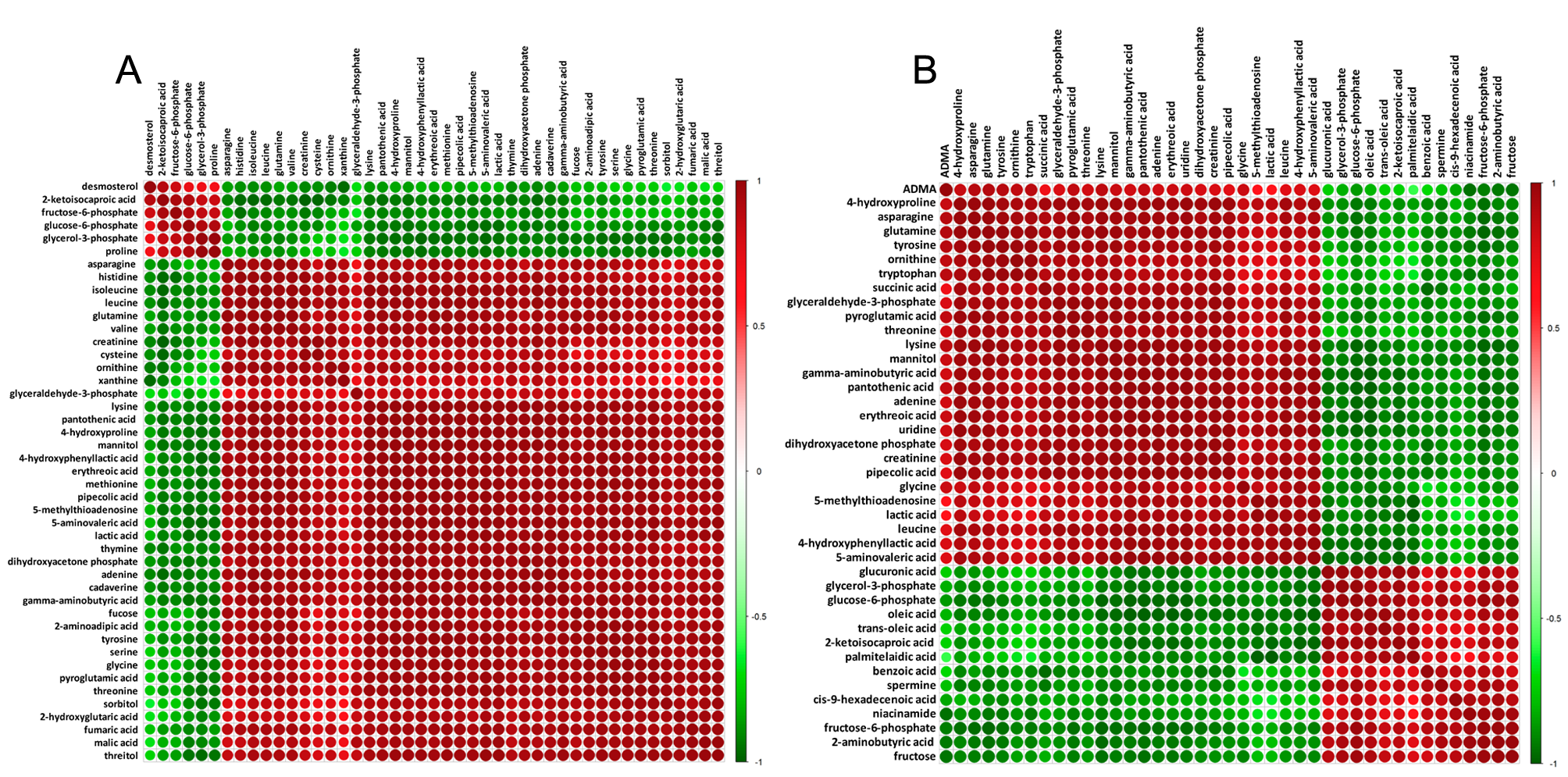


**Figure S3.** Pearson correlation of differentiated metabolites in PK-15 cells infected with the virulent variant (GD-WH) strain PRV at 8 (A) and 16 (B) hpi.


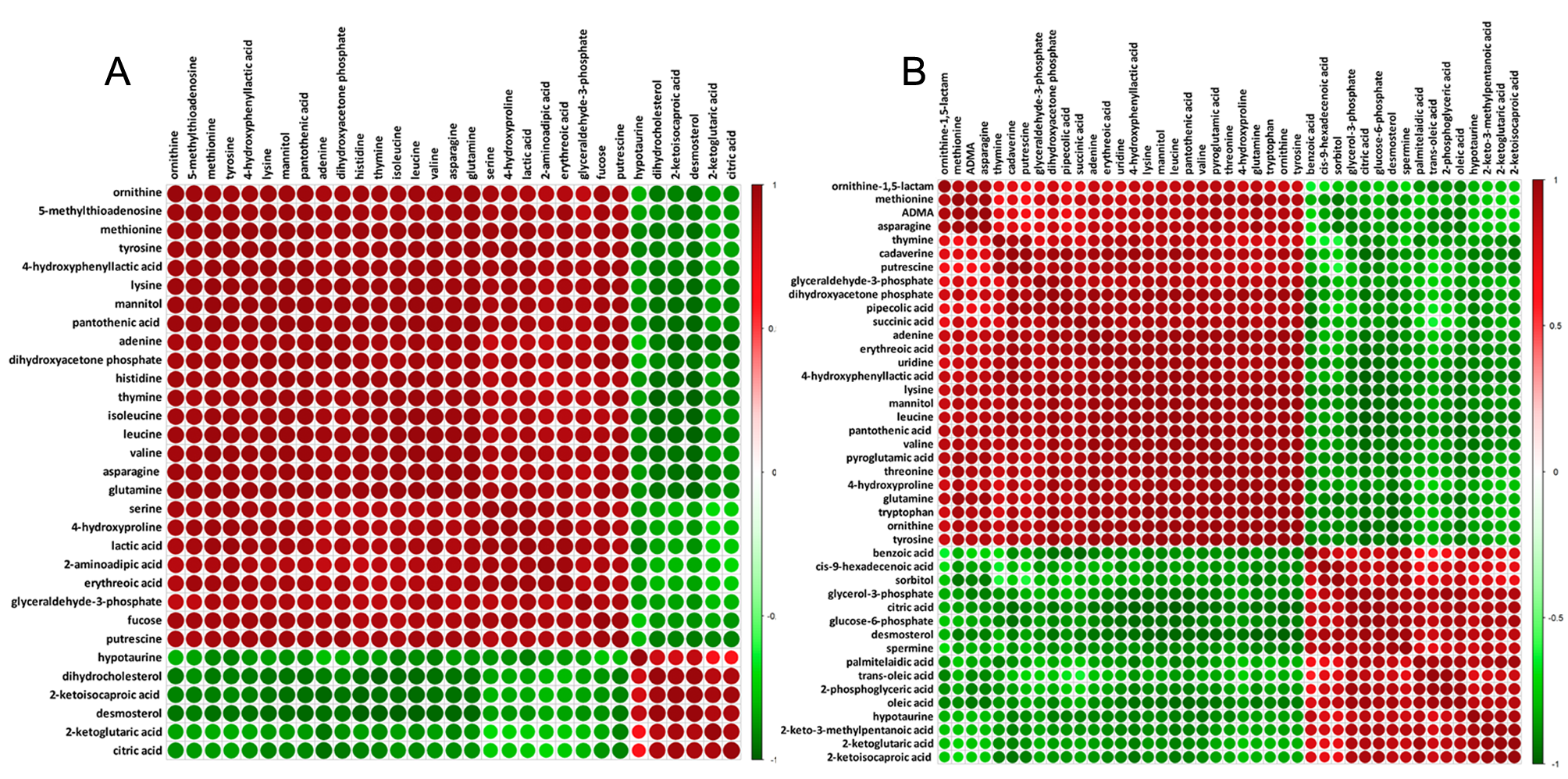


**Figure S4.** Pearson correlation of differentiated metabolites in PK-15 infected with the classical attenuated (Bartha) strain PRV at 8 (A) and 16 (B) hpi.

1.2 Supplementary Tables

**Table S1.** Identification of metabolites in PK-15 cells infected with the virulent variant (GD-WH) strain PRV at 8 hpi

| **Metabolite** | **VIP^a^** | **P-value^b^** | **FC(W8/M8)^c^** | **KEGG** | **Pathway(KEGG)** |
| --- | --- | --- | --- | --- | --- |
| glucose-6p | 1.29 | 7.88E-03 | -1.92 | C01172 | Glycolysis / Gluconeogenesis;  Pentose phosphate pathway |
| fructose-6p | 1.24 | 2.07E-02 | -1.51 | C05345 | Glycolysis / Gluconeogenesis;  Pentose phosphate pathway |
| 2-ketoisocaproic acid | 1.31 | 1.64E-02 | -1.49 | C00233 | Valine, leucine and isoleucine degradation |
| glycerol-3-phosphate | 1.28 | 2.54E-02 | -1.41 | C00093 | Glycerolipid metabolism |
| proline | 1.26 | 2.86E-02 | -1.41 | C00148 | Arginine and proline metabolism |
| desmosterol | 1.19 | 3.83E-02 | -1.37 | C01802 | Steroid biosynthesis |
| 2-oxoglutarate | 1.23 | 4.36E-02 | -2.35 | C03196 | Citrate cycle (TCA cycle) |
| threitol | 1.30 | 1.00E-02 | 1.28 | C16884 |  |
| sorbitol | 1.25 | 2.74E-02 | 1.28 | C00794 | Fructose and mannose metabolism |
| 2-aminoadipic acid | 1.29 | 5.59E-03 | 1.30 | C00956 | Lysine degradation |
| tyrosine | 1.32 | 3.99E-03 | 1.31 | C00082 | Tyrosine metabolism |
| fucose | 1.25 | 6.84E-03 | 1.35 | C01019 | Fructose and mannose metabolism |
| ornithine | 1.27 | 8.91E-03 | 1.37 | C00077 | Arginine and proline metabolism |
| malate | 1.26 | 2.68E-02 | 1.37 | C00149 | Citrate cycle (TCA cycle) |
| pyroglutamic acid | 1.30 | 1.19E-02 | 1.39 | C01879 | Glutathione metabolism |
| fumarate | 1.27 | 1.72E-02 | 1.39 | C00122 | Citrate cycle (TCA cycle) |
| threonine | 1.30 | 5.27E-03 | 1.39 | C00188 | Glycine, serine and threonine metabolism |
| serine | 1.30 | 5.59E-03 | 1.40 | C00065 | Glycine, serine and threonine metabolism |
| glycine | 1.28 | 7.26E-03 | 1.42 | C00037 | Glycine, serine and threonine metabolism |
| methionine | 1.33 | 8.54E-04 | 1.49 | C00073 | Cysteine and methionine metabolism |
| 5-aminovaleric acid | 1.33 | 8.64E-04 | 1.67 | C00431 | Lysine degradation |
| thymine | 1.32 | 2.19E-03 | 1.70 | C00178 | Pyrimidine metabolism |
| glutamine | 1.30 | 5.20E-03 | 1.74 | C00064 | Alanine, aspartate and glutamate metabolism |
| isoleucine | 1.33 | 6.58E-04 | 1.82 | C00407 | Valine, leucine and isoleucine degradation |
| 4-hydroxyproline | 1.34 | 1.52E-04 | 1.84 | C01157 | Arginine and proline metabolism |
| leucine | 1.32 | 8.64E-04 | 1.90 | C00123 | Valine, leucine and isoleucine degradation |
| 4-aminobutyric acid | 1.33 | 3.16E-04 | 1.90 | C00334 | Alanine, aspartate and glutamate metabolism |
| lysine | 1.33 | 2.11E-03 | 1.90 | C00047 | Lysine degradation |
| cysteine | 1.24 | 4.49E-02 | 1.92 | C00097 | Cysteine and methionine metabolism |
| valine | 1.30 | 9.88E-04 | 1.96 | C00183 | Valine, leucine and isoleucine degradation |
| mannitol | 1.34 | 1.65E-04 | 1.99 | C00392 | Fructose and mannose metabolism |
| pipecolic acid | 1.33 | 1.65E-04 | 2.19 | C00408 | Lysine degradation |
| lactic acid | 1.33 | 6.58E-04 | 2.20 | C00186 | Glycolysis / Gluconeogenesis |
| erythreoic acid | 1.33 | 7.48E-04 | 2.24 |  |  |
| 5-methylthioadenosine | 1.33 | 1.02E-03 | 2.33 | C00170 | Cysteine and methionine metabolism |
| asparagine | 1.26 | 1.84E-02 | 2.46 | C00152 | Alanine, aspartate and glutamate metabolism |
| creatinine | 1.29 | 2.40E-02 | 2.47 | C00791 | Arginine and proline metabolism |
| 4-hydroxyphenyllactic acid | 1.33 | 8.64E-04 | 2.54 | C03672 | Tyrosine metabolism |
| adenine | 1.33 | 8.54E-04 | 2.61 | C00147 | Purine metabolism |
| histidine | 1.30 | 2.59E-03 | 2.72 | C00135 | Histidine metabolism |
| xanthine | 1.20 | 4.93E-02 | 2.74 | C00385 | Purine metabolism |
| cadaverine | 1.32 | 6.84E-03 | 2.84 | C01672 | Lysine degradation |
| glyceraldehyde-3p | 1.16 | 4.36E-02 | 3.19 | C00118 | Glycolysis / Gluconeogenesis;  Pentose phosphate pathway |
| glycerone-p | 1.30 | 3.22E-03 | 3.23 | C00111 | Glycolysis / Gluconeogenesis |
| pantothenic acid | 1.34 | 1.65E-04 | 3.56 | C00864 | Pantothenate and CoA biosynthesis |

^a^Variable importance in the projection (VIP) values were obtained from the OPLS-DA model.

^b^The *p* value was analyzed in the “muma” software package in R platform, where parametric test was performed on the data of normal distribution by Welch’s *t* test, while nonparametric test was performed on the data of abnormal distribution by Wilcoxon Mann-Whitney test.

^c^Fold change (FC) was calculated as a binary logarithm of the average mass response (normalized peak area) ratio between PRV-infected groups vs mock groups, where a positive value means that the average mass response of the metabolite in PRV-infected groups is larger than that in mock groups.

**Table S2.** Identification of metabolites in PK-15 cells infected with the virulent variant (GD-WH) strain PRV at 16 hpi

| **Metabolite** | **VIP^a^** | **P-value^b^** | **FC(W16/M16)^c^** | **KEGG** | **Pathway(KEGG)** |
| --- | --- | --- | --- | --- | --- |
| 2-ketoisocaproic acid | 1.26 | 1.31E-02 | -2.75 | C00233 | Valine, leucine and isoleucine degradation |
| niacinamide | 1.21 | 3.56E-02 | -2.18 | C00153 | Nicotinate and nicotinamide metabolism |
| glucose-6p | 1.28 | 5.42E-03 | -9.95 | C01172 | Glycolysis / Gluconeogenesis;  Pentose phosphate pathway |
| fructose-6p | 1.27 | 6.47E-03 | -1.88 | C05345 | Glycolysis / Gluconeogenesis;  Pentose phosphate pathway |
| spermine | 1.21 | 1.89E-02 | -1.81 | C00750 | Arginine and proline metabolism |
| glucuronic acid | 1.25 | 2.68E-02 | -1.76 | C00191 | Pentose and glucuronate interconversions |
| 2-aminobutyric acid | 1.25 | 9.85E-03 | -1.48 | C02356 | Cysteine and methionine metabolism |
| palmitelaidic acid | 1.22 | 2.45E-02 | -1.46 |  |  |
| fructose | 1.26 | 4.48E-03 | -1.46 | C02336 | Amino sugar and nucleotide sugar metabolism |
| oleic acid | 1.28 | 5.10E-04 | -1.45 | C00712 | Fatty acid biosynthesis |
| trans-oleic acid | 1.22 | 1.53E-02 | -1.42 |  |  |
| glycerol-3p | 1.25 | 1.53E-02 | -1.41 | C00093 | Glycerolipid metabolism |
| palmitic acid | 1.14 | 4.08E-02 | -1.40 | C08362 | Fatty acid biosynthesis |
| benzoic acid | 1.18 | 2.71E-02 | -1.32 | C00180 | Benzoate degradation |
| glycine | 1.21 | 1.05E-02 | 1.26 | C00037 | Glycine, serine and threonine metabolism |
| threonine | 1.20 | 1.18E-02 | 1.27 | C00188 | Glycine, serine and threonine metabolism |
| pyroglutamic acid | 1.25 | 3.00E-03 | 1.29 | C01879 | Glutathione metabolism |
| ornithine | 1.16 | 4.39E-02 | 1.39 | C00077 | Arginine and proline metabolism |
| leucine | 1.25 | 1.10E-02 | 1.50 | C00123 | Valine, leucine and isoleucine degradation |
| tyrosine | 1.23 | 2.10E-02 | 1.60 | C00082 | Tyrosine metabolism |
| 4-hydroxyproline | 1.26 | 6.09E-03 | 1.69 | C01157 | Arginine and proline metabolism |
| lactate | 1.23 | 1.17E-02 | 1.75 | C00186 | Glycolysis / Gluconeogenesis |
| succinate | 1.22 | 1.38E-02 | 2.05 | C00042 | Citrate cycle (TCA cycle) |
| tryptophan | 1.19 | 4.89E-02 | 2.06 | C00078 | Tryptophan metabolism |
| pipecolic acid | 1.27 | 7.23E-03 | 2.12 | C00408 | Lysine degradation |
| ADMA(asymmetric dimethylarginine) | 1.16 | 3.85E-02 | 2.13 | C03626 |  |
| 5-methylthioadenosine | 1.22 | 1.09E-02 | 2.20 | C00170 | Cysteine and methionine metabolism |
| 5-aminovaleric acid | 1.28 | 7.73E-04 | 2.30 | C00431 | Lysine degradation |
| glyceraldehyde-3p | 1.23 | 9.40E-03 | 2.44 | C00118 | Glycolysis / Gluconeogenesis;  Pentose phosphate pathway |
| lysine | 1.28 | 9.67E-04 | 2.46 | C00047 | Lysine degradation |
| 4-hydroxyphenyllactic acid | 1.27 | 5.87E-03 | 2.51 | C03672 | Tyrosine metabolism |
| mannitol | 1.27 | 3.00E-03 | 2.59 | C00392 | Fructose and mannose metabolism |
| adenine | 1.28 | 2.02E-03 | 2.62 | C00147 | Purine metabolism |
| glutamine | 1.25 | 8.98E-03 | 2.65 | C00064 | Alanine, aspartate and glutamate metabolism |
| 4-aminobutyric acid | 1.29 | 9.73E-04 | 2.65 | C00334 | Alanine, aspartate and glutamate metabolism |
| erythreoic acid | 1.29 | 2.02E-03 | 2.70 |  |  |
| creatinine | 1.26 | 7.54E-03 | 2.86 | C00791 | Arginine and proline metabolism |
| glycerone-p | 1.25 | 4.08E-03 | 3.10 | C00111 | Glycolysis / Gluconeogenesis |
| asparagine | 1.25 | 5.37E-03 | 4.62 | C00152 | Alanine, aspartate and glutamate metabolism |
| pantothenic acid | 1.29 | 5.10E-04 | 5.38 | C00864 | Pantothenate and CoA biosynthesis |
| uridine | 1.29 | 5.42E-03 | +∞ | C00299 | Pyrimidine metabolism |

^a^Variable importance in the projection (VIP) values were obtained from the OPLS-DA model.

^b^The *p* value was analyzed in the “muma” software package in R platform, where parametric test was performed on the data of normal distribution by Welch’s *t* test, while nonparametric test was performed on the data of abnormal distribution by Wilcoxon Mann-Whitney test.

^c^Fold change (FC) was calculated as a binary logarithm of the average mass response (normalized peak area) ratio between PRV-infected groups vs mock groups, where a positive value means that the average mass response of the metabolite in PRV-infected groups is larger than that in mock groups.

**Table S3.** Identification of metabolites in PK-15 cells infected with the classical attenuated (Bartha) strain PRV at 8 hpi

| **Metabolite** | **VIP^a^** | **P-value^b^** | **FC(B8/M8)^c^** | **KEGG** | **Pathway(KEGG)** |
| --- | --- | --- | --- | --- | --- |
| desmosterol | 1.28 | 1.02E-02 | -2.34 | C01802 | Steroid biosynthesis |
| hypotaurine | 1.21 | 2.34E-02 | -1.72 | C00519 | Taurine and hypotaurine metabolism |
| citrate | 1.22 | 1.87E-02 | -1.84 | C00158 | Citrate cycle (TCA cycle) |
| 2-ketoisocaproic acid | 1.26 | 1.25E-02 | -1.47 | C00233 | Valine, leucine and isoleucine degradation |
| dihydrocholesterol | 1.26 | 8.22E-03 | -1.43 |  |  |
| 2-oxoglutarate | 1.16 | 3.55E-02 | -1.41 | C00026 | Citrate cycle (TCA cycle) |
| 2-aminoadipic acid | 1.20 | 3.51E-02 | 1.19 | C00956 | Lysine degradation |
| 4-hydroxyproline | 1.26 | 2.38E-02 | 1.20 | C01157 | Arginine and proline metabolism |
| serine | 1.24 | 2.38E-02 | 1.30 | C00065 | Glycine, serine and threonine metabolism |
| erythreoic acid | 1.23 | 1.50E-02 | 1.31 |  |  |
| thymine | 1.28 | 7.10E-03 | 1.39 | C00178 | Pyrimidine metabolism |
| tyrosine | 1.29 | 6.08E-03 | 1.39 | C00082 | Tyrosine metabolism |
| putrescine | 1.26 | 7.10E-03 | 1.42 | C00134 | Arginine and proline metabolism |
| ornithine | 1.26 | 7.10E-03 | 1.43 | C00077 | Arginine and proline metabolism |
| 5-methylthioadenosine | 1.26 | 8.36E-03 | 1.47 | C00170 | Cysteine and methionine metabolism |
| fucose | 1.24 | 1.02E-02 | 1.48 | C01019 | Fructose and mannose metabolism |
| lactate | 1.26 | 3.37E-02 | 1.48 | C00186 | Glycolysis / Gluconeogenesis |
| methionine | 1.30 | 1.06E-03 | 1.56 | C00073 | Cysteine and methionine metabolism |
| adenine | 1.26 | 7.08E-03 | 1.60 | C00147 | Purine metabolism |
| glutamine | 1.29 | 2.21E-04 | 1.69 | C00064 | Alanine, aspartate and glutamate metabolism |
| isoleucine | 1.29 | 2.47E-04 | 1.82 | C00407 | Valine, leucine and isoleucine degradation |
| lysine | 1.29 | 2.47E-03 | 2.04 | C00047 | Lysine degradation |
| leucine | 1.29 | 3.63E-03 | 2.05 | C00123 | Valine, leucine and isoleucine degradation |
| mannitol | 1.30 | 1.05E-04 | 2.18 | C00392 | Fructose and mannose metabolism |
| valine | 1.30 | 9.06E-04 | 2.45 | C00183 | Valine, leucine and isoleucine degradation |
| asparagine | 1.28 | 7.28E-04 | 2.55 | C00152 | Alanine, aspartate and glutamate metabolism |
| 4-hydroxyphenyllactic acid | 1.30 | 2.99E-04 | 3.01 | C03672 | Tyrosine metabolism |
| histidine | 1.29 | 4.41E-04 | 3.32 | C00135 | Histidine metabolism |
| pantothenic acid | 1.30 | 1.16E-04 | 3.40 | C00864 | Pantothenate and CoA biosynthesis |
| glyceraldehyde-3p | 1.20 | 1.36E-02 | 3.86 | C00118 | Glycolysis / Gluconeogenesis;  Pentose phosphate pathway |
| glycerone-p | 1.28 | 8.43E-03 | 4.92 | C00111 | Glycolysis / Gluconeogenesis |

^a^Variable importance in the projection (VIP) values were obtained from the OPLS-DA model.

^b^The *p* value was analyzed in the “muma” software package in R platform, where parametric test was performed on the data of normal distribution by Welch’s *t* test, while nonparametric test was performed on the data of abnormal distribution by Wilcoxon Mann-Whitney test.

^c^Fold change (FC) was calculated as a binary logarithm of the average mass response (normalized peak area) ratio between PRV-infected groups vs mock groups, where a positive value means that the average mass response of the metabolite in PRV-infected groups is larger than that in mock groups.

**Table S4.** Identification of metabolites in PK-15 cells infected with the classical attenuated (Bartha) strain PRV at 16 hpi

| **Metabolite** | **VIP^a^** | **P-value^b^** | **FC(B16/M16)^c^** | **KEGG** | **Pathway(KEGG)** |
| --- | --- | --- | --- | --- | --- |
| hypotaurine | 1.22 | 3.86E-02 | -2.81 | C00519 | Taurine and hypotaurine metabolism |
| citrate | 1.30 | 2.75E-03 | -2.67 | C00158 | Citrate cycle (TCA cycle) |
| desmosterol | 1.29 | 8.68E-03 | -2.63 | C01802 | Steroid biosynthesis |
| 2-oxoglutarate | 1.22 | 4.88E-02 | -1.96 | C00026 | Citrate cycle (TCA cycle) |
| 2-keto-3-methylpentanoic acid | 1.24 | 3.25E-02 | -1.93 | C00671 | Valine, leucine and isoleucine degradation |
| glucose-6p | 1.27 | 1.27E-02 | -1.86 | C01172 | Glycolysis / Gluconeogenesis;  Pentose phosphate pathway |
| spermine | 1.22 | 3.25E-02 | -1.74 | C00750 | Arginine and proline metabolism |
| 2-ketoisocaproic acid | 1.24 | 3.36E-02 | -1.69 | C00233 | Valine, leucine and isoleucine degradation |
| glycerol-3p | 1.25 | 4.79E-03 | -1.58 | C00093 | Glycerolipid metabolism |
| palmitic acid | 1.20 | 2.46E-02 | -1.57 | C08362 | Fatty acid biosynthesis |
| 2-phosphoglyceric acid | 1.20 | 9.81E-03 | -1.53 | C00631 | Glycolysis / Gluconeogenesis;  Pentose phosphate pathway |
| oleic acid | 1.26 | 4.79E-03 | -1.48 | C00712 | Fatty acid biosynthesis |
| palmitelaidic acid | 1.21 | 3.19E-02 | -1.46 |  |  |
| trans-oleic acid | 1.12 | 3.06E-02 | -1.39 |  |  |
| benzoic acid | 1.20 | 2.46E-02 | -1.35 | C00180 | Benzoate degradation |
| sorbitol | 1.19 | 3.25E-02 | -1.21 | C00794 | Fructose and mannose metabolism |
| 4-hydroxyproline | 1.26 | 8.84E-03 | 1.34 | C01157 | Arginine and proline metabolism |
| thymine | 1.22 | 3.04E-02 | 1.35 | C00178 | Pyrimidine metabolism |
| pyroglutamic acid | 1.29 | 8.49E-04 | 1.36 | C01879 | Glutathione metabolism |
| threonine | 1.28 | 7.56E-03 | 1.43 | C00188 | Glycine, serine and threonine metabolism |
| putrescine | 1.23 | 2.33E-02 | 1.44 | C00134 | Arginine and proline metabolism |
| erythreoic acid | 1.28 | 1.42E-02 | 1.45 |  |  |
| pipecolic acid | 1.23 | 2.04E-02 | 1.55 | C00408 | Lysine degradation |
| valine | 1.30 | 2.15E-04 | 1.78 | C00183 | Valine, leucine and isoleucine degradation |
| adenine | 1.28 | 5.40E-03 | 1.83 | C00147 | Purine metabolism |
| succinate | 1.23 | 1.10E-02 | 1.92 | C00042 | Citrate cycle (TCA cycle) |
| leucine | 1.31 | 3.84E-05 | 2.05 | C00123 | Valine, leucine and isoleucine degradation |
| ornithine | 1.29 | 7.12E-03 | 2.05 | C00077 | Arginine and proline metabolism |
| tyrosine | 1.29 | 7.16E-03 | 2.13 | C00082 | Tyrosine metabolism |
| cadaverine | 1.28 | 8.68E-03 | 2.16 | C01672 | Lysine degradation |
| methionine | 1.21 | 1.94E-02 | 2.23 | C00073 | Cysteine and methionine metabolism |
| glyceraldehyde-3p | 1.21 | 3.25E-02 | 2.62 | C00118 | Glycolysis / Gluconeogenesis;  Pentose phosphate pathway |
| lysine | 1.31 | 1.57E-04 | 3.18 | C00047 | Lysine degradation |
| tryptophan | 1.29 | 1.14E-02 | 3.29 | C00078 | Tryptophan metabolism |
| glutamine | 1.28 | 4.16E-03 | 3.35 | C00064 | Alanine, aspartate and glutamate metabolism |
| mannitol | 1.31 | 5.55E-05 | 3.46 | C00392 | Fructose and mannose metabolism |
| 4-hydroxyphenyllactic acid | 1.30 | 7.74E-04 | 3.75 | C03672 | Tyrosine metabolism |
| ornithine-1,5-lactam | 1.10 | 3.78E-02 | 3.91 |  |  |
| glycerone-p | 1.28 | 2.41E-03 | 3.92 | C00111 | Glycolysis / Gluconeogenesis |
| ADMA(asymmetric dimethylarginine) | 1.18 | 3.53E-02 | 4.53 | C03626 |  |
| asparagine | 1.19 | 2.75E-02 | 5.00 | C00152 | Alanine, aspartate and glutamate metabolism |
| pantothenic acid | 1.31 | 1.08E-04 | 5.40 | C00864 | Pantothenate and CoA biosynthesis |
| uridine | 1.29 | 1.18E-02 | +∞ | C00299 | Pyrimidine metabolism |

^a^Variable importance in the projection (VIP) values were obtained from the OPLS-DA model.

^b^The *p* value was analyzed in the “muma” software package in R platform, where parametric test was performed on the data of normal distribution by Welch’s *t* test, while nonparametric test was performed on the data of abnormal distribution by Wilcoxon Mann-Whitney test.

^c^Fold change (FC) was calculated as a binary logarithm of the average mass response (normalized peak area) ratio between PRV-infected groups vs mock groups, where a positive value means that the average mass response of the metabolite in PRV-infected groups is larger than that in mock groups.
